# When Psychosis Is Just the Beginning: Acute Intermittent Porphyria Presenting as a First-Episode Psychosis

**DOI:** 10.1192/j.eurpsy.2026.11038

**Published:** 2026-07-31

**Authors:** I. Ramos-Suarez, C. Quesada

**Affiliations:** Psichiatry, Hospital Universitario San Cecilio, Granada, Spain

## Abstract

**Introduction:**

Acute Intermittent Porphyria (AIP) is a rare autosomal dominant disorder of heme biosynthesis. Although it typically presents with abdominal pain, autonomic instability, and motor neuropathy, psychiatric symptoms—especially acute psychosis—can occasionally precede the somatic signs, leading to misdiagnosis and treatment delays.

**Objectives:**

To illustrate the diagnostic challenges posed by atypical presentations of AIP, and to emphasize the importance of considering metabolic etiologies in first-episode psychosis with evolving neurological symptoms.

**Methods:**

We describe the clinical course of a 44-year-old male with no prior psychiatric history, admitted to a psychiatric ward for suspected first-episode psychosis. During hospitalization, the patient developed progressive motor weakness and autonomic instability. Neurological assessment, laboratory testing (including urinary porphobilinogen), and clinical evolution led to the final diagnosis.

**Results:**

The patient initially presented with psychotic symptoms, including agitation, disorganized thinking, and paranoid delusions. Within days, he developed ascending muscle weakness and gait disturbances, followed by autonomic signs (tachycardia, hypertension), requiring transfer to the Intensive Care Unit. Elevated urinary porphobilinogen confirmed AIP. He received intravenous hemin and supportive treatment, leading to a favorable outcome with full remission of both neurological and psychiatric symptoms.

**Conclusions:**

AIP should be included in the differential diagnosis of acute psychosis, especially when accompanied by emerging neuromuscular or autonomic symptoms.

- Early identification and treatment are crucial to prevent severe complications.
- This case reinforces the value of a multidisciplinary approach in atypical psychiatric presentations.

**Disclosure of Interest:**

None Declared

